# Supplementary material for: Clinical Evaluation of a Fully-Automated High-Throughput Multiplex Screening-Assay to Detect and Differentiate the SARS-CoV-2 B.1.1.529 (Omicron) and B.1.617.2 (Delta) Lineage Variants
Source: Viruses. 2022 Mar 15;14(3):608. doi: 10.3390/v14030608 (PMC8950896; doi:10.3390/v14030608)
Supplement: Supplementary file 1 [file viruses-14-00608-s001.zip › viruses-1570188-supplementary.pdf]

Nörz et al.

Clinical evaluation of a fully-automated high-throughput multiplex screening-assay to detect and differentiate the SARS-CoV-2 B.1.1.529 (Omicron) and B.1.617.2 (Delta) lineage variants.

Supplementary figures

Supplementary Figure S1

RBD-484-fwd : RBD-484-fwd

Delta G: -9.28 kcal/mole **Base Pairs:** 4

```
5'  CTATCAGGCCGGTARCA
     :  : |||| :  :
3' ACRATGGCCGGACTATC
```

RBD-484-fwd : RBD-484-fwd

Delta G: -9.75 kcal/mole **Base Pairs:** 4

```
5' CTATCAGGCCGGTARCA
      :    ||||    :
3'    ACRATGGCCGGACTATC
```

P681-fwd : P681-fwd

Delta G: -9.89 kcal/mole **Base Pairs:** 4

```
5' TGCAGGTATATGCGCTAGTTA
          :   ||||   :
3'      ATTGATCGCGTATATGGACGT
```

RBD-484-fwd : P681R-probe

Delta G: -9.82 kcal/mole **Base Pairs:** 4

```
5' CTATCAGGCCGGTARCA
          ||||   : :
3'       GCGGCTGCTCTTA
```

RBD-484-fwd : P681H-probe

Delta G: -9.82 kcal/mole **Base Pairs:** 4

```
5' CTATCAGGCCGGTARCA
          |||| : : :
3'       GCGGCTACTCTGA
```

Oligo dimer alignments were generated using OligoAnalyzer Software (Integrated DNA Technologies, USA). Multiple consequtive matches at the 3' ends of both oligos are considered especially risky.

Supplementary Figure S2

Target: SDEL2

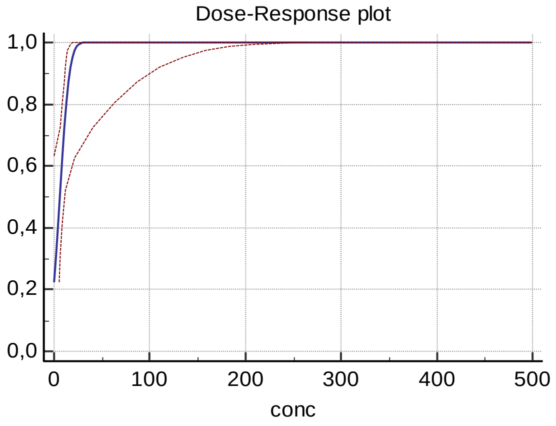

19.0 IU/ml (CI95%: 12.9 – 132.2 IU/ml)

Target: E484A

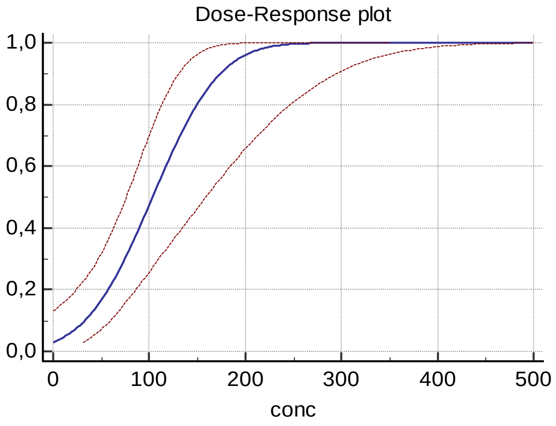

193.9 IU/ml (CI95%: 144.7 – 334.7 IU/ml)

Target: P681H

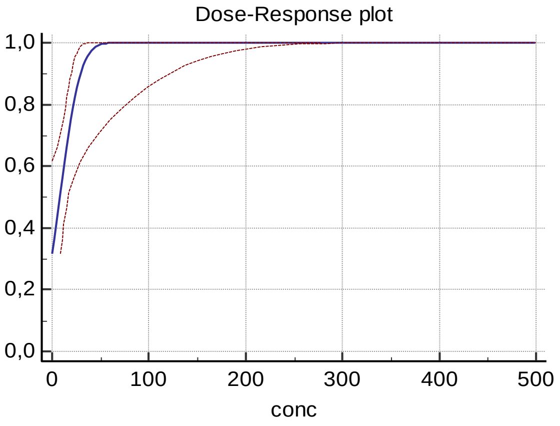

35.5 IU/ml (CI95%: 23.3 – 158.0 IU/ml)

Target: P681R

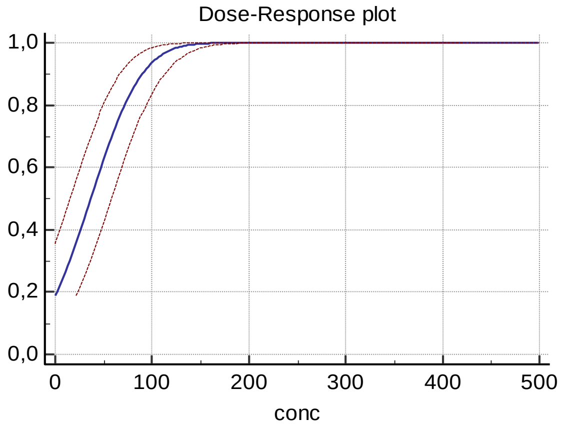

104.99 IU/ml (CI95%: 80.74 – 129.25 IU/ml)

Probit plots for LoD experiments were generated using MedCalc Software (Ostend, Belgium).

Supplementary Figure S3

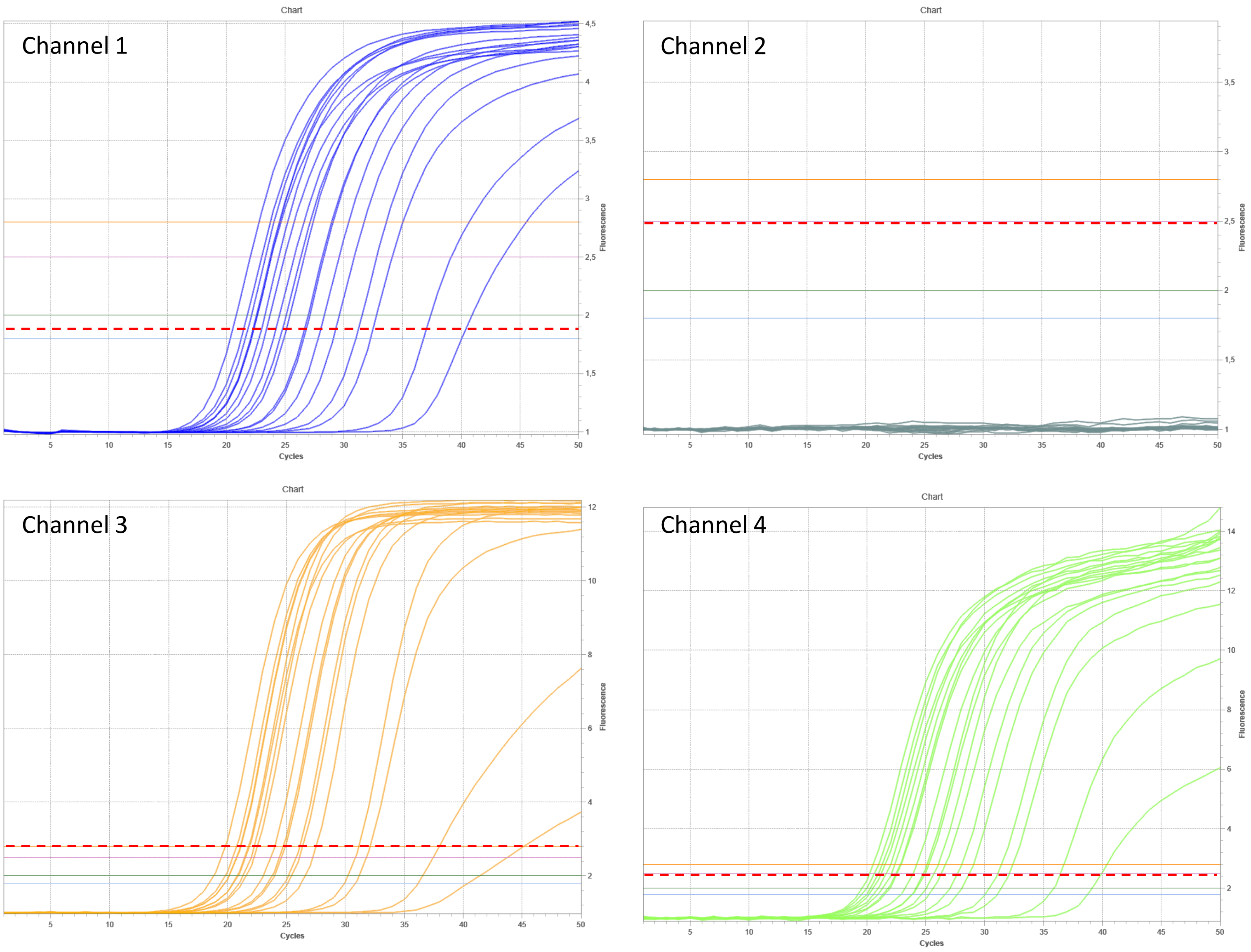

*Amplification curves of **BA.1-like** lineage (**Omicron variant**) samples, taken from the clinical sample set. Images were exported from the Utility Channel Optimization tool (Roche), Thresholds are indicated as dotted red line. (For exact threshold values, see table 2)*

Supplementary Figure S4

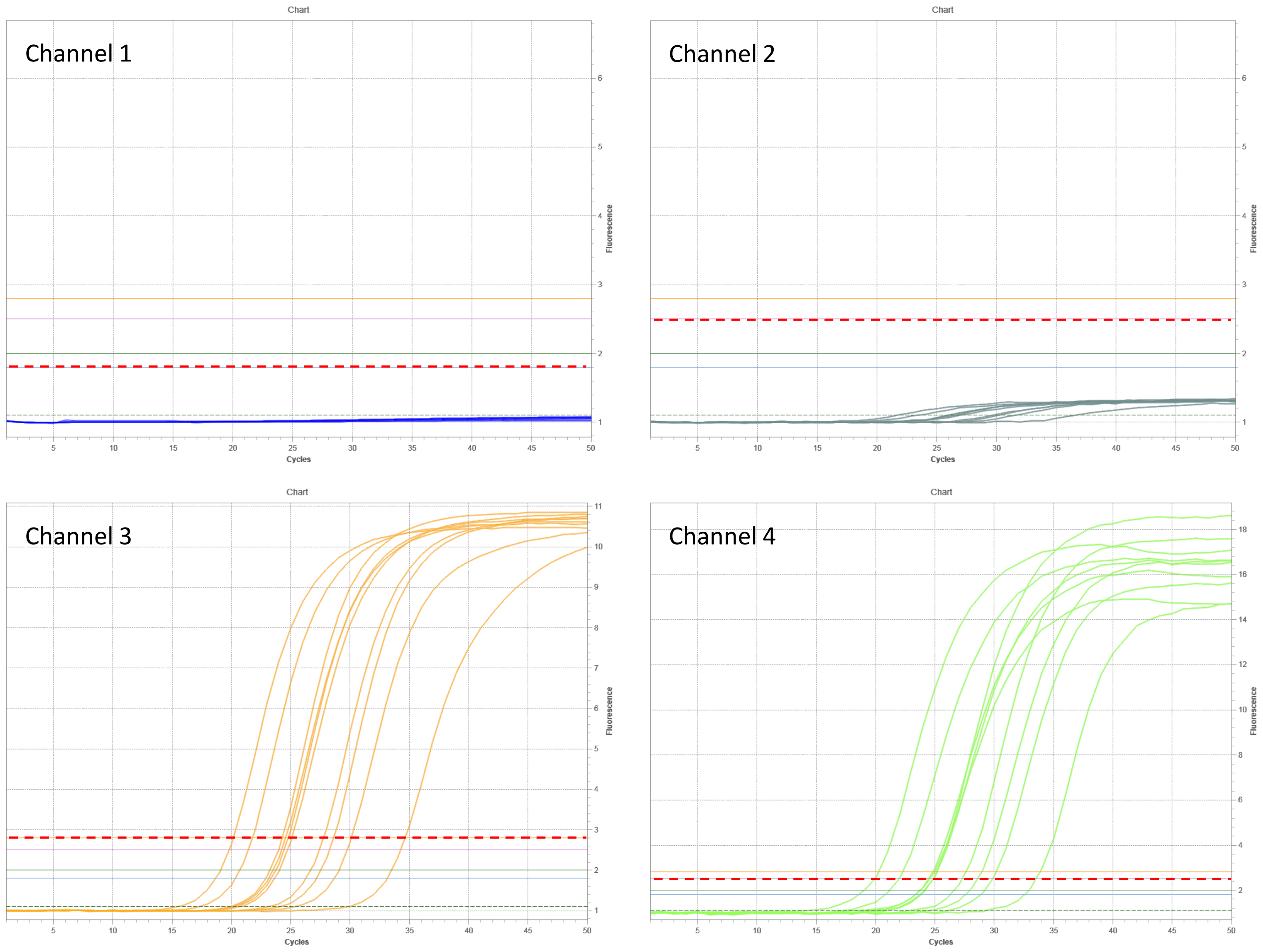

Amplification curves of ***BA.2-like*** *lineage* ***(Omicron variant)*** *samples, taken from the clinical sample set. Images were exported from the Utility Channel Optimization tool (Roche), Thresholds are indicated as dotted red line. (For exact threshold values, see table 2)*

Supplementary Figure S5

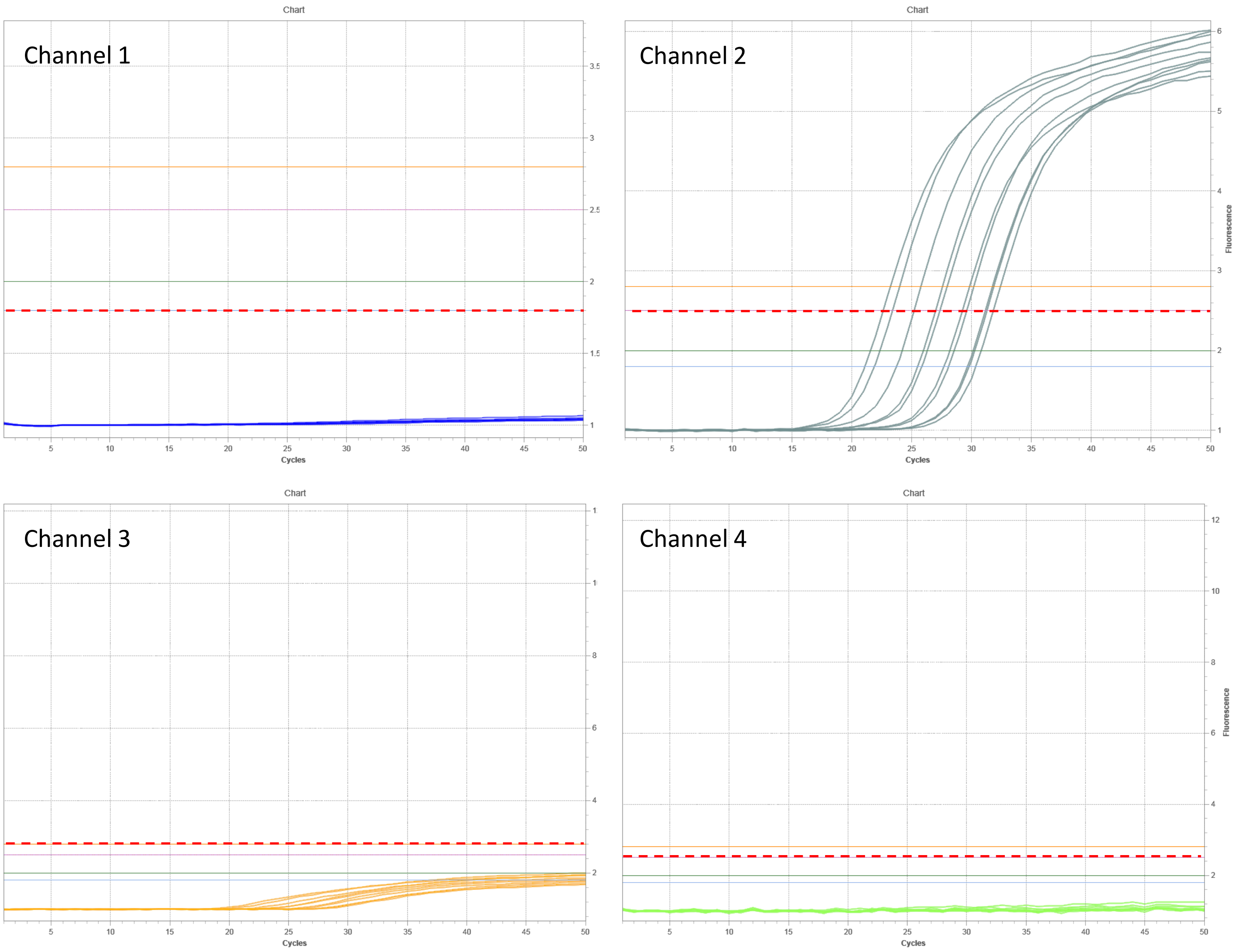

Amplification curves of ***B.1.617.2*** *lineage (****Delta variant****) samples, taken from the clinical sample set. Images were exported from the Utility Channel Optimization tool (Roche), Thresholds are indicated as dotted red line. (For exact threshold values, see table 2)*

Supplementary Figure S6

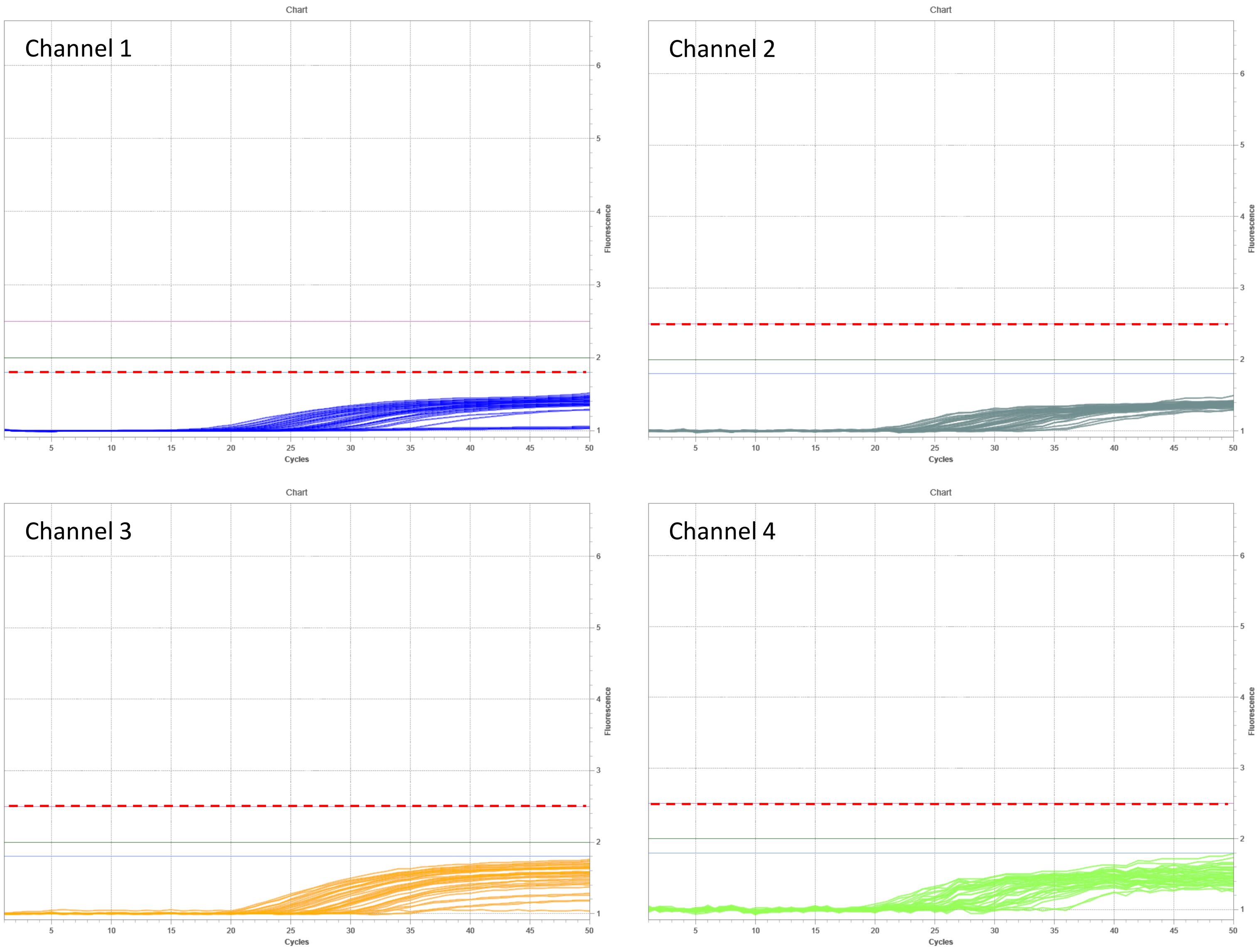

*Amplification curves of **B.1.1.7** lineage (**Alpha variant**) samples, taken from the clinical sample set. Images were exported from the Utility Channel Optimization tool (Roche), Thresholds are indicated as dotted red line. (For exact threshold values, see table 2)*

Nörz et al.

Clinical evaluation of a fully-automated high-throughput multiplex screening-assay to detect and differentiate the SARS-CoV-2 B.1.1.529 (Omicron) and B.1.617.2 (Delta) lineage variants.

Supplementary tables

Supplementary Table S1

Targets:	1		3	4		2	
Relevant mutations:	A67V	del-HV69-70	E484A	N679K	P681H	P681R	Detected by one or more multiplex targets?
BA.1 (Omicron variant)	x	x	x	x	x		yes
BA.2 (Omicron variant)			x	x	x		yes
B.1.617.2 (Delta variant)			(x)			x	yes
B.1.1.7 (Alpha variant)		x			x		no
B.1.351 (Beta variant)							no
P.1 (Gamma variant)							no

Overview of current or former VOCs and the distribution of mutations relevant for the multiplex assay. Note that the E484A SNP does rarely occur in the Delta variant, but would return a positive result if present. For target 1 and target 4, both sequence variations need to be present in order to generate a positive result.

Supplementary Table S2

	Total	Perfect align	%
d67_70	13462	13346	99.14%
E484A	3472	3424	98.62%
P681H	11910	11870	99.66%
P681R	11910	11	0.09%
All 3 targets	2984	2982	99.93%

(i.e. at least one target positive)

All available Omicron variant sequences were analyzed by Roche Diagnostics (Pleasanton, USA) for missmatches with oligos used in this study. The number of valid sequences in the respective regions are listed. The value for all 3 targets represents the portion of sequences that are a prefect match for at least one of the three Omicron variant specific assays.

Supplementary Table S3

Primer	NTD F	NTD R	Probe1	RBD F	484 F	484 R	RBD R	Probe3	681 F	681 R	Probe4	Probe2
Zhen F	3.90	3.90	5.99	4.74	5.97	4.25	6.84	5.24	4.88	3.90	4.52	2.92
Zhen R	3.90	3.29	7.30	3.40	4.97	5.00	3.90	3.90	3.29	5.00	2.92	2.92
Zhen P V4	5.99	7.30	5.02	5.02	4.01	4.64	6.62	5.24	4.25	3.43	4.75	4.75
RBD F	5.24	3.90	5.02	5.74	4.97	3.90	6.08	1.95	2.91	3.90	3.05	3.42
484 F	5.24	4.25	4.01	4.74	9.75	6.68	6.69	5.24	5.70	6.21	9.82	9.82
484 R	4.25	5.00	4.64	5.13	6.68	4.62	4.88	3.29	5.85	5.38	5.00	3.61
RBD R	6.84	3.90	6.62	6.08	5.97	4.88	7.05	7.05	7.05	7.04	3.43	5.12
484A P	5.24	3.90	5.24	1.95	5.97	3.29	7.05	7.05	7.05	7.04	3.43	3.14
681 F	4.88	3.29	4.25	3.79	4.97	5.85	7.05	7.05	9.89	5.09	6.75	6.75
681 R	3.90	5.00	3.43	3.42	6.21	5.38	7.04	7.04	5.09	3.43	5.00	3.42
681H P	4.52	2.92	4.75	3.05	9.82	5.00	3.43	3.43	6.75	5.00	3.61	3.61
681R P	2.92	2.92	4.75	3.42	9.82	3.61	5.12	3.14	6.75	3.42	3.61	3.61

Every oligo in the assay set was checked for dimerization with every other oligo in the set. Oligo sequences were entered in OligoAnalyzer Software (Integrated DNA Technologies, USA) and the highest binding energy constellation entered in the table (All values represent negative energy differential). Values above 8 (i.e. -8 kcal/mole) were further analyzed for overlap at the 3′ ends, see supplementary Figure S1.

Supplementary Table S4

Primer	NTD F	NTD R	Probe1	RBD F	484 F	484 R	RBD R	Probe3	681 F	681 R	Probe4	Probe2
Zhen F	0	0	0	0	0	0	0	0	0	0	0	0
Zhen R	0	0	0	0	0	0	0	0	0	0	0	0
Zhen P	0	0	0	0	0	0	0	0	0	0	0	0
RBD F	0	0	0	0	0	0	0	0	0	0	0	0
484 F	0	0	0	0	0	0	0	0	0	0	0	0
484 R	0	0	0	0	0	0	0	0	0	0	0	0
RBD R	0	0	0	0	0	0	969	0	0	0	0	0
484 P	0	0	0	0	0	0	0	57	0	0	0	0
681 F	0	0	0	0	0	0	0	0	0	672	0	0
681 R	0	0	0	0	0	0	0	0	0	0	0	0
681H P	0	0	0	0	0	0	0	0	0	0	0	0
681R P	0	0	0	0	0	0	0	0	0	0	0	0

Database: RefSeq RNA

RBD R: Homo sapiens GLI family zinc finger 2 (GLI2) (mRNA)

484P: Homo sapiens translation initiation factor IF-2 (LOC105371191) (ncRNA)
Homo sapiens paraneoplastic antigen Ma6E-like (LOC105379547)

681R: Homo sapiens nucleoporin 188 (NUP188) (mRNA)

Every oligo combination was analyzed for potential amplification of human DNA or RNA sequences. Values in the table represent amplificate length in bp. Amplified targets are listed below. Probes are not considered relevant for production of unspecific amplificates. Products of 300 bp and below are a high risk of efficient amplification.

Supplementary Table S5

External Quality Control Panel

Species	Number tested	Target: SDEL2	Target: P681R	Target: E484A	Target: P681H
Influenza A H1N1 pdm09 (A/California/60/2008)	1	Negative	Negative	Negative	Negative
Influenza B Victoria (B/Brisbane/60/2008)	1	Negative	Negative	Negative	Negative
RSV-A	1	Negative	Negative	Negative	Negative
RSV-B	1	Negative	Negative	Negative	Negative
MERS-CoV	1	Negative	Negative	Negative	Negative
hCoV-229E (ZeptoMetrix)	1	Negative	Negative	Negative	Negative
Parainfluenza-1 (ZeptoMetrix)	1	Negative	Negative	Negative	Negative
Parainfluenza-4 (ZeptoMetrix)	1	Negative	Negative	Negative	Negative
SARS-CoV (Frankfurt-1, cell culture supernatant)	1	Negative	Negative	Negative	Negative

Clinical samples

Species		Target: SDEL2	Target: P681R	Target: E484A	Target: P681H
hCoV - HKU1	1	Negative	Negative	Negative	Negative
hCoV - NL63	1	Negative	Negative	Negative	Negative
RSV	3	Negative	Negative	Negative	Negative
Adenovirus	3	Negative	Negative	Negative	Negative
Boca-virus	3	Negative	Negative	Negative	Negative
Parainfluenzavirus 3	3	Negative	Negative	Negative	Negative
Human Metapneumovirus	2	Negative	Negative	Negative	Negative
Rhino-/Enterovirus	2	Negative	Negative	Negative	Negative

Viral species tested as part of the exclusivity/cross-reactivity panel. Clinical samples or external control material was utilized based on availability.
